# Supplementary material for: Conditional chemoconnectomics (cCCTomics) as a strategy for efficient and conditional targeting of chemical transmission
Source: eLife. 2024 Apr 30;12:RP91927. doi: 10.7554/eLife.91927 (PMC11060718; doi:10.7554/eLife.91927)
Supplement: Figure 3—source data 3. [file elife-91927-fig3-data3.zip › Figure 3 source data3 part 1.zip!/Figure 3-Source data 3-part1/1071-1-CRISPResso_Report_osfbjvga.zip!/CRISPResso_on_1071_2/4d.Position_dependent_average_indel_size.pdf]

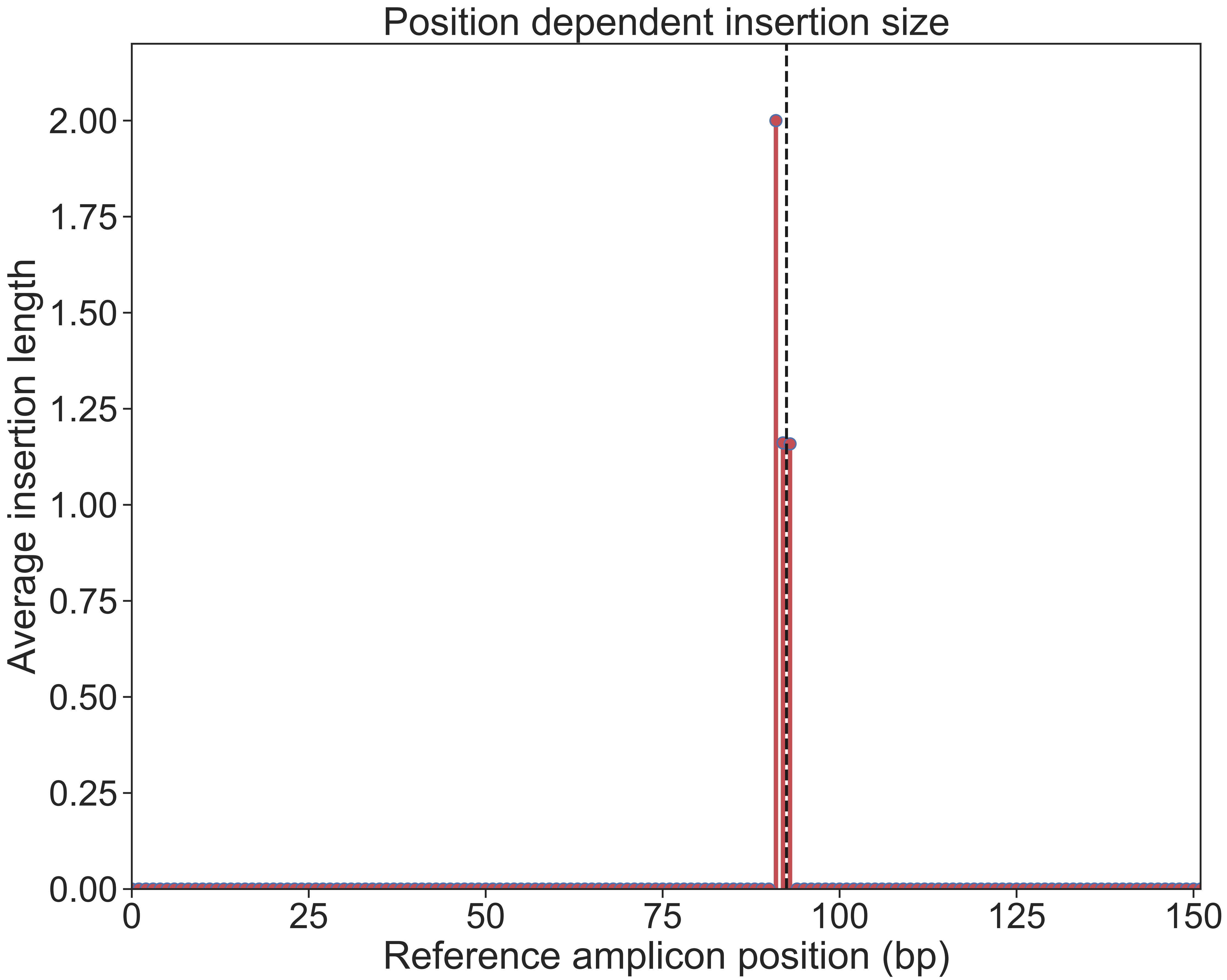

Position dependent insertion size
Average insertion length
2.00
1.75
1.50
1.25
1.00
0.75
0.50
0.25
0.00
0
25
50
75
100
125
150
Reference amplicon position (bp)

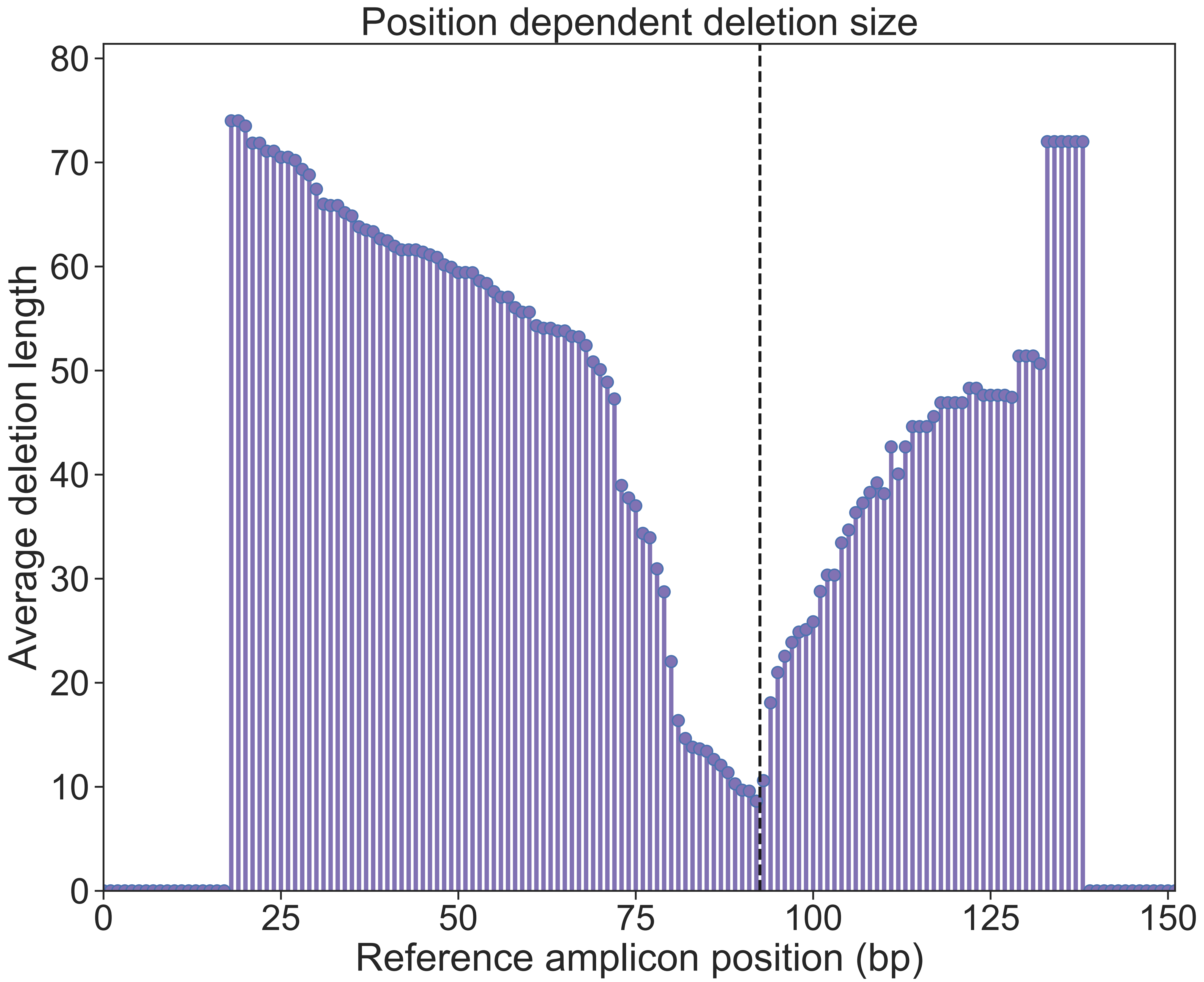

Position dependent deletion size
Average deletion length
80
70
60
50
40
30
20
10
0
0
25
50
75
100
125
150
Reference amplicon position (bp)
